# Role of bovine herpesvirus type 5 (BoHV-5) in diseases of cattle. Recent findings on BoHV-5 association with genital disease

**Published:** 2012-05-13

**Authors:** P.A. Favier, M.S. Marin, S.E. Pérez

**Affiliations:** 1 *Becaria Agencia Nacional de Promoción Científica y Tecnológica (ANPCyT)- FONCyT, Facultad de Ciencias Veterinarias, Universidad Nacional del Centro de la Provincia de Buenos Aires (UNCPBA), Paraje Arroyo Seco S/N. Tandil (7000), Argentina*; 2 *Facultad de Ciencias Veterinarias, Universidad Nacional del Centro de la Provincia de Buenos Aires (UNCPBA), Paraje Arroyo Seco S/N. Tandil (7000), Argentina*; 3 *Comisión Nacional de Investigaciones Científicas y Técnicas (CONICET). Avenida Rivadavia 1917. Buenos Aires (C1033AAJ), Argentina*; 4 *Instituto Nacional de Tecnología Agropecuaria (INTA) Balcarce. Departamento de Producción Animal. Ruta 226, km 73,5. Balcarce (7600), Argentina*

**Keywords:** BoHV-5, Reproductive disorders, Semen

## Abstract

Bovine herpesvirus type 5 (BoHV-5) belongs to the family *Herpesviridae*, subfamily *Alphaherpesvirinae*, genus *Varicellovirus*. This virus is a major causative agent of non-suppurative meningoencephalitis in young cattle. It was first isolated in 1962 from a neurological disease outbreak in Australia. BoHV-5 is genetically and antigenically related to bovine herpesvirus type 1 (BoHV-1), a highly prevalent virus responsible for respiratory and genital disease in cattle. Initially, BoHV-5 was considered a subtype of BoHV-1 (BoHV-1.3). However, the exclusive presentation of outbreaks of neurological disease suggested that the virus was a new agent with characteristics of neuropathogenicity. Even though both are neurotropic viruses, only BoHV-5 is capable of replicating extensively in the central nervous system and inducing neurological disease. Occasionally, encephalitis caused by BoHV-1 has been reported. Like other alpha-herpesviruses, BoHV-5 can establish latency in nervous ganglia and, by stress factors or glucocorticoid treatment, latent virus can be reactivated. During episodes of reactivation, the virus is excreted in nasal, ocular and genital secretions and transmitted to other susceptible hosts. Recently, BoHV-5 has been associated with infection of the reproductive tract. The virus has been isolated and the presence of viral DNA has been demonstrated in semen samples from Brazil and Australia and natural transmission of the virus through contaminated semen has also been described. Embryos and oocytes are permissive for BoHV-5 infection and BoHV-5 DNA has been detected in the central nervous system of aborted fetuses. The objective of this review is to compile the limited information on the recent association between BoHV-5 and reproductive disorders in cattle.

## Introduction

Bovine herpesvirus type 5 (BoHV-5) is a major causative agent of non-suppurative meningoencephalitis in young cattle (Magyar *et al.*, 1993; Schwyzer and Ackermann, 1996; Pérez *et al.*, 2002; Delhon *et al.*, 2003; Del Médico Zajac *et al.*, 2010). The virus was first isolated in 1962 from a neurological disease outbreak in Australia (Johnston *et al.*, 1962).

Thereafter, the virus has been sporadically identified in cases of meningoencephalitis in this country (Studdert, 1989; Kirkland *et al.*, 2009), in the United States (d´Offay *et al.*, 1993; Ely *et al.*, 1996) and in Europe (Abdelmagid *et al.*, 1995). However, the disease has a high incidence in South American countries, mainly in Central and Southern Brazil and Argentina (Pérez *et al.*, 2002; Vogel *et al.*, 2003). The reasons for this restricted geographical distribution of neurological cases are unknown.

BoHV-5 is genetically and antigenically related to bovine herpesvirus type 1 (BoHV-1), a virus responsible for a variety of clinical syndromes, including respiratory disease, conjunctivitis, abortion and genital infections (Tikoo *et al.*, 1995; Jones and Chowdhury, 2007).

After natural or experimental infections, alpha-herpesviruses establish latent infections in sensory neurons within trigeminal or dorsal root ganglia (Stevens and Cook, 1971). Viral reactivation from latency can occur naturally, by stress factors, or it can be experimentally induced by glucocorticoid administration. During episodes of reactivation, the virus is re-excreted in nasal, ocular and genital secretions and transmitted to other susceptible hosts (Rock *et al.*, 1992). This is the main mechanism by which alpha-herpesviruses are maintained in nature.

Initially, BoHV-5 was considered a subtype of BoHV-1 (BoHV-1.3) (Engels *et al.*, 1986; Metzler *et al.*, 1986; Studdert, 1989). However, the exclusive presentation of outbreaks of neurological disease suggested that the virus was a new agent with characteristics of neuropathogenicity (Moretti *et al.*, 1964; Watt *et al.*, 1981; Weiblen *et al.*, 1989).

Both BoHV-1 and BoHV-5 are neurotropic viruses (Meyer *et al.*, 2001; Pérez *et al.*, 2002; Varela *et al.*, 2010). Nevertheless, both differ in the ability to cause encephalitis (Ashbaugh *et al.*, 1997). Even though encephalitis caused by BoHV-1 has been reported (Meyer *et al.*, 2001), only BoHV-5 is capable of extensive replication in the central nervous system (CNS) and production of neurological disease (Delhon *et al.*, 2003).

In 1992, the International Committee on Taxonomy of Viruses recognized BoHV-5 as a distinct virus from BoHV-1(Roizman, 1992; Pedraza and Alessi, 2004). The decision was based on the results of restriction site mapping of viral DNA (d´Offay *et al.*, 1993; Whetstone *et al.*, 1993), cross-neutralization studies, and reactivity with monoclonal antibodies (Metzler *et al.*, 1986; Collins *et al.*, 1993).

Presently, strains of BoHV-5 are designated as subtypes “a”, “b” and “non a-non b”, according to restriction endonuclease patterns (D’Arce *et al.*, 2002; Maidana *et al.*, 2011). The transient presence of subtype “b” strains has only been described in Argentina. Nevertheless, in this country, the most common circulating BoHV-5 strains belong to subtype “a” (Maidana *et al.*, 2011).

Further studies are required to classify additional field strains and to compare with the profile of BoHV-5 variants circulating in other countries.

## Properties of BoHV-5

BoHV-5 belongs to the family *Herpesviridae*, subfamily *Alphaherpesvirinae*, genus *Varicellovirus*. The virion is enveloped and its diameter can range from 120 to 250 nm, containing an icosahedral nucleocapsid composed by 162 capsomers (Knowles, 2011). The nucleocapsid is surrounded by a proteinaceous layer, called tegument (Thiry *et al.*, 2006).

The genome consists of a single linear molecule of double-stranded DNA and it is classified as a type D genome, which consists of a unique long (UL) and a unique short (US) region, flanked by repeated sequences (Thiry *et al.*, 2006). The genome of BoHV-5 (138, 390 base pairs) is longer than BoHV-1genome and it contains 72 genes (Delhon *et al.*, 2003). Both viruses share 85% genetic similarity (Chowdhury, 1995) and approximately 82% amino acid identity (Delhon *et al.*, 2003).

The lytic replicative cycle of alpha-herpesviruses occurs in a regulated cascade. A virion component activates expression of immediate-early (IE) genes, which have regulatory roles in the activation of early (E) and late (L) genes. In latently infected neurons, the latency-related (LR) gene, a small region of BoHV genome is abundantly transcribed (Rock *et al.*, 1987; Kutish *et al.*, 1990; Schang and Jones, 1997). BoHV-5 -IE proteins (bICP0, bICP4 and bICP22) are the least conserved proteins. The LR gene, which has essential roles in the latency-reactivation cycle of alpha-herpesviruses (Jones, 2003), differs markedly from its BoHV-1 counterpart (Delhon *et al.*, 2003), suggesting that this condition may have important consequences on the *in vivo* virus pathogenesis.

In cell cultures, BoHV-1 and BoHV-5 share similar morphology and cytopathic effect. Unlike BoHV-1, BoHV-5 is difficult to isolate in cell culture (Carrillo, 1982; Belknap *et al.*, 1994; d´Offay *et al.*, 1995; Ely *et al.*, 1996; Cascio *et al.*, 1999), even from animals experimentally inoculated and showing neurological signs. The difficulty in virus isolation has been related to the low viral titers in tissues samples (Belknap *et al.*, 1994).

Traditionally, BoHV-1 and BoHV-5 are differentiated based on the clinical features of the outbreaks and on DNA restriction profiles after virus isolation (Afonso *et al.*, 2007). More recently, the development of specific PCR techniques has allowed the precise differentiation of both viruses in clinical samples (van Engelenburg *et al.*, 1993, 1995; Chowdhury, 1995; Diallo *et al.*, 2010).

## Neurological disease associated with BoHV-5

Outbreaks of meningoencephalitis caused by BoHV-5 are commonly observed in South American countries (Pérez *et al.*, 2002; Vogel *et al.*, 2003). The prevalence of BoHV-5 is presently unknown, mainly because of the serological cross-reactivity with BoHV-1(Vogel *et al.*, 2002; Oldoni *et al.*, 2004; Diel *et al.*, 2007).

The first outbreak of BoHV-5 in Argentina was described in 1982 (Carrillo, 1982). However, in a retrospective study of Argentinean cases with histopathological diagnosis of cerebrocortical necrosis between 1970 and 1999, Pérez *et al*. (2003) demonstrated by *in situ* hybridization that BoHV-5 DNA was present in some archival sections of neural tissues. Results from this study confirmed that BoHV-5 was circulating in the country long before the virus was recognized as a causative agent of neurological disorder in cattle.

BoHV-1 pathogenesis has been studied in detail and many aspects can be extrapolated to BoHV-5. The respiratory tract is the main portal of entry of BoHV into the organism. Initial viral replication in the epithelium of the upper respiratory tract and in the tonsils may lead to slight respiratory signs (Meyer *et al.*, 2001; Vogel *et al.*, 2004). Primary replication at the site of entry may last for 15 days (Vogel *et al.*, 2004). In the case of BoHV-5, after replication at mucosal sites, the virus reaches the CNS where viral replication causes neurological damage (Beltrão *et al.*, 2000, Meyer *et al.*, 2001, Pérez *et al.*, 2002).

Two main routes have been described for the access of BoHV-5 to the CNS, 1) via the olfactory tract, through the terminals of the axons that innervate the olfactory mucosa (Nakazato *et al.*, 2000; Pérez *et al.*, 2002) and, 2) via trigeminal nerve, reaching the sensory neurons of trigeminal ganglia where latency is established (Chowdhury *et al.*, 1997; Lee *et al.*, 1999; Coelho and Vidor, 2001; Pérez *et al.*, 2002, 2005).

Unlike BoHV-1, latency of BoHV-5 in other sites of CNS is also possible (Meyer *et al.*, 2001). It has been suggested that viral reactivation from latency may lead to virus spread to different areas of the brain and this would be responsible for the presence of mild neurological signs during episodes of reactivation (Vogel *et al.*, 2003). Neurological signs begin 9 to 10 days post-infection and they include tremors, depression, opistothonus, nystagmus, teeth gridding, circling and recumbency (Carrillo *et al.*, 1983; Beltrão *et al.*, 2000; Meyer *et al.*, 2001, Pérez *et al.*, 2002; Vogel *et al.*, 2003). Signs are progressive and usually fatal (Belknap *et al.*, 1994; Beltrão *et al.*, 2000). BoHV-5 has been considered a classical neurotropic herpesvirus.

However, Schudel *et al*. (1986) demonstrated that the virus was not restricted to the CNS of animals with clinical signs since the virus was isolated, in parallel, from the respiratory tract. Moreover, in this study, BoHV-5 was recovered from an aborted fetus from a farm in which sporadic cases of abortion were recorded but no cases of neurological disease had been observed. Thus, it is apparent that the virus it not only confined to the respiratory tract and CNS. This is the first presumptive association of BoHV-5 with clinical syndromes different from neurological disease.

## Genital disease associated with BoHV-1 and recent findings on reproductive disorders associated with BoHV-5

BoHV-1 isolates are classified into two subtypes; BoHV-1.1, which is mainly responsible for bovine infectious rhinotracheitis, and BoHV-1.2, which is regularly isolated from cases of genital disease (Engels *et al.*, 1986).

The biological differences between subtypes are not clear. Although both subtypes are able to infect the respiratory and genital tracts, it is apparent that each genotype has specific adaptations for growing in particular tissues. Rijsewijk *et al*. (1999) suggested that differences in glycoprotein C may be responsible for the distinct phenotypes.

Infections of the reproductive tract are usually caused and disseminated by artificial insemination (AI) (Turin *et al.*, 1999). Genital disorders associated with BoHV-1 include pustular balanoposthitis and vulvovaginitis. Balanoposthitis is usually accompanied by a reduction in semen quality (van Oirschot, 1995). It is known that insemination of cows with BoHV-1-contaminated semen can also cause abortion, infertility and endometritis (Elazhary *et al.*, 1980) and it has been demonstrated that the virus dose required to infect the cow via contaminated semen is extremely low, approximately 32 infectious viral particles (Bielanski *et al.*, 1988; Turin *et al.*, 1999). Although abortions might occur as a consequence of genital disease, they are more frequently observed after BoHV-1 respiratory outbreaks (Turin *et al.*, 1999).

In the bull, the virus replicates in the mucosa of the prepuce, penis and urethra after entering the organism via the respiratory or genital tract (van Oirschot, 1995). Contamination of the semen occurs during ejaculation from virus present in the mucosa of the reproductive tract (van Engelenburg *et al.*, 1995). Thus, BoHV-1 DNA is detected in the seminal fluid fraction (van Engelenburg *et al.*, 1993; van Oirschot, 1995).

After genital infection, BoHV-1 establishes latency in sacral ganglia and virus reactivation is a potential source for semen contamination (Ackermann and Wyler, 1984). It is well-established that BoHV-1-latently infected cattle can excrete virus, intermittently, in semen (van Engelenburg *et al.*, 1993, 1995). Therefore, sero-positive bulls should not be allowed in AI centers. Nevertheless, Lemaire *et al*. (2000) also reported the existence of sero-negative latent carriers, which are an epidemiological threat for AI centers. Furthermore, BoHV-1 is detected in semen during primary infection, before the onset of clinical signs (Weiblen *et al.*, 1992). Therefore, animals without evidence of clinical disease are also a potential source of venereal virus transmission.

Recently, there have been several reports describing the detection of BoHV-5 in bull semen. Gomes *et al*. (2003) showed that 6 out of 20 (30%) semen samples, previously considered positive for the presence of BoHV-1 were, indeed, BoHV-5-positive, when re-evaluated by a BoHV-5-specific PCR.

In Brazil, a semen sample from an apparently healthy bull, which was identified as BoHV-1-positive during virus surveillance at an AI center in 1996 was re-analyzed by Esteves *et al*. (2003). In this work, the isolate previously characterized as BoHV-1 was re-classified as BoHV-5. The identity of the virus was determined by the use of BoHV-5-specific monoclonal antibodies in an immunoperoxidase assay and by a gC BoHV-1/5 PCR.

The first evidence of natural transmission of BoHV-5 through contaminated semen was described by Kirkland *et al*. (2009), in Australia. Later, in the same country, Diallo *et al*. (2010) isolated the virus from cryopreserved semen of a healthy bull. The identity of the virus was confirmed by PCR and by direct amplicon sequencing, demonstrating the importance of using molecular techniques for the detection of viral agents in semen samples. In Brazil, Oliveira *et al*. (2011) used a two species-specific nested PCR which differentiates BoHV-1 and BoHV-5 and they identified BoHV-5 DNA in 100% (76/76) semen samples. Additionally, in 44.7% (34/76) samples, BoHV-1 DNA was also present.

Kirkland *et al*. (2009) described an outbreak of vulvovaginitis with clinical signs and gross lesions identical to those caused by BoHV-1. After virus isolation, BoHV-1 was considered the presumptive causal agent of the genital infection. Later, by restriction enzyme analysis and quantitative PCR it was determined that BoHV-5 was the virus detected in the semen of the bull used for AI. Therefore, it was suggested that BoHV-5 was responsible for the outbreak of vulvovaginitis previously attributed to BoHV-1.

Silva-Frade *et al*. (2010b) documented the detection of BoHV-5 DNA in the acrosomal region and tail of spermatozoids experimentally exposed to the virus. Nevertheless, it has not been elucidated whether the virus infects the spermatozoid or it is attached to its surface. Among the sperm parameters, the velocity of spermatozoid progression seems to be reduced and a low level of apoptosis, without affecting the total quality of the sperm, has also been reported (Souza *et al.*, 2011). Apparently, unlike BoHV-1 (Tanghe *et al.*, 2005), experimental BoHV-5 infection of spermatozoids does not interfere with *in vitro* fertilization or embryo development (Souza *et al.*, 2011).

Silva-Frade *et al*. (2010a) demonstrated that oocytes and embryos were infected by BoHV-5 when experimentally exposed to the virus. By *in situ* hybridization it was shown that the virus was present in the cumulus cells of the oocytes and in the inner cells of the embryos. BoHV-5 decreased the cleavage rate of oocytes only when they were grown in medium containing fetal bovine serum. However, adverse effects were not observed when polyvinyl alcohol medium was used. Thus, the effect on cleavage rate seems to be dependent on other conditions rather than BoHV-5 infection alone. Viral replication in oocytes and embryos was proved by an increase in virus titers from an inoculation dose of 10^2.3^ TCID_50_/50μl to 10^5.3^TCID_50_/50μl at the embryo stage (Silva-Frade *et al.*, 2010b), demonstrating that these cells are permissive for BoHV-5 infection.

Abortions are a common sequel of BoHV-1- respiratory infection. However, the role of BoHV-5 in abortion has not been definitively demonstrated. Recently, in Argentina, Marin *et al*. (2011) identified BoHV-5 in the CNS of aborted fetuses. Virus isolation from fetal spleen and brain on MDBK cells, a multiplex PCR and seroneutralization tests for BoHV-1 and 5 were carried out. BoHV was not isolated. Nevertheless, BoHV-5 DNA-specific sequences were detected in fetal nervous tissue by multiplex PCR and confirmed by sequencing of PCR products. The presence of BoHV-5 DNA in aborted fetal nervous system is the first description of this virus in association with bovine abortion.

The use of molecular biology techniques for the identification of BoHV-5 in this study was essential to improve the sensitivity of the diagnosis of infectious causes of bovine abortion, especially when autolysis was present. It also allowed the differentiation of the type of BoHV involved. Although virus DNA detection does not prove BoHV-5 is responsible for the abortion, its presence in fetal tissues is suggestive of its involvement.

Many aspects of BoHV-5 association with genital disease remain to be elucidated. It is likely that the pathogenesis of this disorder is similar to that of BoHV-1.

However, further studies are required to establish the role of the virus in the infection of the reproductive tract. Furthermore, other issues need to be addressed. For example, it has not been established whether the infection of a cow with contaminated semen can lead to a latent infection or whether infection by this route can cause neurological disease.

Batches of frozen semen from bulls of accredited AI centers are normally certified negative for acute epidemic diseases.

Presently, an increasing number of these centers are also certified negative for viruses like bovine leukemia virus (BLV), BoHV-1 and bovine viral diarrhea virus (BVDV) (Wrathall *et al.*, 2006). The precise role of BoHV-5 in reproductive disease of cattle is still under study. However, the sole presence of the virus in bovine semen should be considered as a threat for the international trade of frozen semen. As a consequence, detection of BoHV-5 should be included in the pathogen screening policies of AI centers.

## Conclusion

For decades, BoHV-5 has been considered a viral agent with exclusive tropism for the CNS. However, recent reports have involved BoHV-5 in the development of genital disease. Presently, the studies on the role of BoHV-5 in diseases of the reproductive tract are not conclusive.

However, there are many evidences that demonstrate that the virus is a potential threat for AI centers since it is excreted in semen and it can be transmitted to susceptible cows. To prevent the spread of the virus by AI, all semen samples, even those of apparently healthy bulls, should be analyzed. Further studies are required to establish the precise role of BoHV-5 in bovine abortion and other conditions of the genital tract.

Furthermore, this review highlights the importance of the application of molecular biology techniques for the characterization and differentiation of bovine herpesviruses in clinical samples.
